# Integrated ITS2 DNA Barcoding and LC–QTOF/MS Metabolomics for Authentication of Ngueak Pla Mo (*Acanthus* spp.) and Differentiation of Pharmacopoeial Species from *A. montanus* L.

**DOI:** 10.3390/plants15152349

**Published:** 2026-07-30

**Authors:** Siripat Chaichit, Ampai Phrutivorapongkul, Warunya Arunotayanun, Aekkhaluck Intharuksa

**Affiliations:** 1 Department of Pharmaceutical Sciences, Faculty of Pharmacy, Chiang Mai University, Chiang Mai 50200, Thailand; siripat.chaichit@cmu.ac.th (S.C.); ampai.phrutiv@cmu.ac.th (A.P.); 2 Kanchanabhishek Institute of Medical and Public Health Technology, Faculty of Public Health and Allied Health Science, Praboromarajchanok Institute, Nonthaburi 11150, Thailand; warunya@kmpht.ac.th

**Keywords:** *Acanthus*, Ngueak Pla Mo, herbal authentication, DNA barcoding, ITS2, LC–QTOF/MS, metabolomics, quality control

## Abstract

Ngueak Pla Mo is a Thai medicinal crude drug derived from the pharmacopoeial *Acanthus* species *A. ebracteatus* Vahl and *A. ilicifolius* L.; however, morphological similarity and the occurrence of non-pharmacopoeial substitutes such as *A. montanus* (Nees) T. Anderson may complicate authentication and quality control. This study aimed to develop an integrated molecular–chemical approach for differentiating pharmacopoeial Ngueak Pla Mo species from *A. montanus*. Twenty authenticated *Acanthus* samples were analyzed using ITS2 DNA barcoding, including nucleotide variation, pairwise genetic distance, barcode gap, haplotype network, phylogenetic analysis, and predicted ITS2 secondary structure. Representative samples were further examined by untargeted LC–QTOF/MS metabolite profiling combined with hierarchical clustering, PCA, PLS-DA, VIP analyses, fold-change evaluation, and specificity scoring. ITS2 analyses clearly separated *A. montanus* from *A. ebracteatus* and *A. ilicifolius*, supported by diagnostic sequence variation, positive barcode gaps, distinct haplotypes, phylogenetic clustering, and secondary-structure differences, whereas *A. ebracteatus* and *A. ilicifolius* could not be reliably differentiated from each other at the species level using ITS2. LC–QTOF/MS profiling revealed species-consistent metabolite patterns and prioritized gallic acid, catechol, 3,4-dihydroxybenzoic acid, trigonelline, and ursolic acid as preliminary candidate markers for *A. montanus* discrimination. These results indicate that combining ITS2 barcoding with metabolite profiling provides complementary evidence for Ngueak Pla Mo authentication and supports future quality-control development.

## 1. Introduction

**Figure 1:**
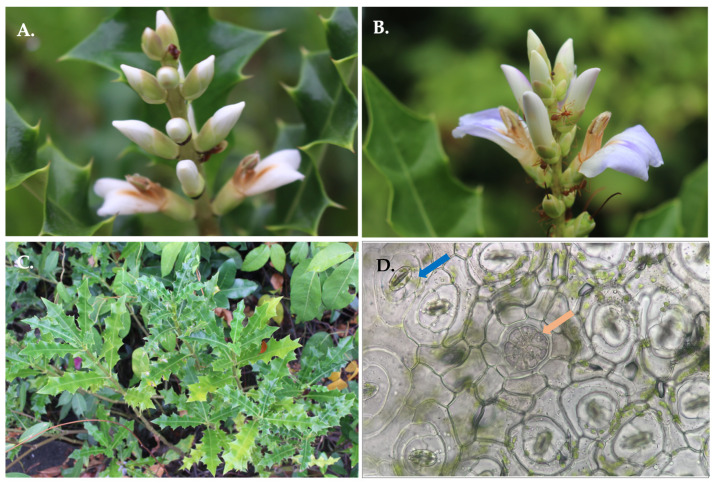
Morphological and micromorphological characteristics of *Acanthus* species used as Ngueak Pla Mo. (**A**) Inflorescence and flower morphology of *Acanthus ebracteatus*; (**B**) inflorescence and flower morphology of *Acanthus ilicifolius*; (**C**) habit and leaf morphology of *Acanthus* species; and (**D**) leaf epidermal micromorphology showing diacytic stomata (blue arrow) and salt gland (orange arrow). (Photos taken by Aekkhaluck Intharuksa).

Ngueak Pla Mo is a Thai medicinal crude drug obtained from mangrove-associated species of *Acanthus* L. (Acanthaceae) [1]. According to the Thai Herbal Pharmacopoeia, two species are officially recognized as botanical sources: *Acanthus ebracteatus* Vahl (Acanthi Ebracteati Herba) [2] (Figure 1A) and *Acanthus ilicifolius* L. (Acanthi Ilicifolii Herba) [3] (Figure 1B). Both are prepared from the dried whole plant and are indicated for topical anti-inflammatory use [2,3], while *A. ebracteatus* is additionally recognized for antipruritic applications [2]. Both species are spiny shrubs (Figure 1C) occurring in coastal or mangrove habitats and possess thick, glossy, opposite leaves with dentate or spiny margins. *A. ebracteatus* and *A. ilicifolius* share similar morphological features, and diagnostic microscopic characters, including salt glands (Figure 1D), diacytic stomata (Figure 1D), sclereids, vessels, fibers, and crystals, but they can be differentiated by flower color (Figure 1A,B). The anatomical features are important for pharmacognostic authentication to distinguish it from other plant species, particularly when the crude drug is dried or powdered. Phytochemical studies across multiple *Acanthus* species (e.g., *A. ilicifolius*, *A. ebracteatus*, *A. montanus*, *A. mollis* and *A. hirsutus*) have revealed a chemically diverse profile of specialized metabolites, including phenolic and phenylpropanoid glycosides, flavonoids, iridoid glycosides, lignan glycosides, triterpenoids and other terpenoids, steroids and alkaloids, with saponins also being reported in some species [4,5,6,7,8]. Verbascoside, also known as acteoside, is one of the major phenylethanoid glycosides reported in both *A. ebracteatus* and *A. ilicifolius* and has been used as a reference compound for chromatographic identification [2,3,8,9]. UPLC-ESI-QTOF-MS and UPLC analyses have revealed that *A. ebracteatus* extracts are rich in phenolic acids, including hydroxybenzoic (e.g., gallic, protocatechuic) and hydroxycinnamic acids (e.g., chlorogenic, caffeic, ferulic, sinapic and rosmarinic acids) [7,10]. In contrast, phytochemical studies on *A. ilicifolius* have revealed a broad spectrum of secondary metabolites, including flavonoids, alkaloids, lignans, terpenoids, various glycosides, steroids, phenylethyl alcohol derivatives (phenylethanols), coumarins, and benzoxazinoid-related compounds [5,8,11]. The traditional use of Ngueak Pla Mo is supported by pharmacological evidence demonstrating antioxidant, anti-inflammatory, antimicrobial, wound-healing, photoprotective, anti-aging, neuroprotective, hepatoprotective, and antidiabetic activities. Verbascoside-rich extracts of *A. ebracteatus* have demonstrated promising skin-related bioactivities, including the promotion of fibroblast-mediated wound closure, protection of keratinocyte–fibroblast cocultures from UV-induced photodamage, and significant suppression of UV-stimulated MMP secretion, effects that are consistent with the antioxidant and anti-inflammatory properties of verbascoside [10]. Meanwhile, *A. ilicifolius* has been associated with broad pharmacological properties, including antioxidant, anti-inflammatory, antimicrobial, anticancer, hepatoprotective, gastroprotective, anti-arthritic, and antidiabetic effects [8,12].

Despite their medicinal importance, the quality control of Ngueak Pla Mo remains challenging because this crude drug name refers to more than one closely related *Acanthus* species. Their morphological similarity, overlapping traditional uses, and use of the same vernacular name may lead to misidentification or substitution, especially in dried, fragmented, powdered, or processed materials [13,14]. This issue is further complicated by our preliminary market investigation, which revealed the presence of *A. montanus* (Nees) T. Anderson as a potential substitute or misidentified material sold under the name Ngueak Pla Mo. Unlike the official Thai Herbal Pharmacopoeia species, *A. ebracteatus* and *A. ilicifolius*, *A. montanus* is not recognized as an authentic botanical source of this crude drug. *A. montanus*, commonly known as mountain bear’s breeches, is a shrubby perennial of the family Acanthaceae, widely cultivated as an ornamental plant and traditionally used medicinally in West and Central Africa [15]. The occurrence of this non-pharmacopoeial and alien *Acanthus* species in herbal materials raises important concerns regarding species authenticity, chemical consistency, therapeutic reliability, and regulatory quality assurance.

The potential substitution of official Ngueak Pla Mo species with *A. montanus* underscores the need for a more reliable and comprehensive authentication strategy. Although macroscopic and microscopic examinations are essential for pharmacognostic evaluation, these methods may be insufficient when diagnostic organs are absent or when samples are dried, fragmented, or powdered [16,17]. DNA barcoding has emerged as a useful molecular approach for herbal authentication because it enables species identification based on standardized genetic regions and can detect substitution or adulteration even when morphological characters are no longer visible [18,19,20]. Among plant barcode regions, the nuclear ribosomal internal transcribed spacer 2 (ITS2) has been widely recognized as a suitable marker for medicinal plant identification because of its short length, high amplification efficiency, sequence variability, and strong species-level discriminatory power [21]. Large-scale validation and subsequent reviews have supported the use of ITS2 as an effective barcode for identifying medicinal plants and closely related species [22,23,24,25]. In Acanthaceae, ITS2 has been reported as the most suitable marker among *mat*K, *rbc*L, *trn*H–*psb*A, *trn*L, and ITS2 for Bar-HRM-based discrimination of Thai medicinal Acanthaceae species [26]. Using ITS2-derived primers, previous work successfully discriminated dried samples of 16 medicinal Acanthaceae species at both genus and species levels, supporting the value of ITS2 for authentication of processed herbal materials in this family [26]. Therefore, ITS2 represents a promising barcode candidate for discriminating the official Ngueak Pla Mo species, *A. ebracteatus* and *A. ilicifolius*, from the non-pharmacopoeial species *A. montanus*. Nevertheless, although DNA-based authentication provides reliable taxonomic evidence for species identification, its application can be limited when DNA is highly degraded, fragmented, or removed during herbal processing, particularly in extensively dried, heat-treated, powdered, or extracted materials [27,28]. In addition, DNA barcoding cannot directly evaluate chemical composition, bioactive marker abundance, or pharmacologically relevant metabolite variation among samples [29]. Chemical analysis is therefore required as a complementary approach to assess the phytochemical quality and consistency of herbal materials.

Chemical profiling is an important analytical strategy for the quality control of herbal materials, as it allows the characterization of complex phytochemical patterns and supports the assessment of marker compounds, sample consistency, adulteration, and quality-related variation influenced by environmental and post-harvest factors [30,31]. To complement molecular authentication, chemical profiling was therefore employed to characterize phytochemical variation and to assess the consistency of quality-related metabolites among Ngueak Pla Mo materials. LC–QTOF/MS has become an important platform for medicinal plant quality control because it provides high-resolution accurate mass detection and broad metabolite coverage, allowing comprehensive characterization of specialized metabolites in complex herbal matrices [32,33]. Previous phytochemical investigations have demonstrated that *Acanthus* species contain diverse metabolites, including phenolic glycosides, flavonoids, iridoids, lignans, terpenoids, and related phenolic compounds [7]. Among these, phenylethanoid glycosides, particularly verbascoside, are important constituents reported in *A. ebracteatus* and *A. ilicifolius* and are relevant to pharmacopoeial identification of Ngueak Pla Mo. However, comparative LC–QTOF/MS-based profiling among the official Ngueak Pla Mo species and potential substitutes remains limited. Therefore, this study aimed to develop an integrated authentication strategy for Ngueak Pla Mo by combining ITS2–based DNA barcoding and LC–QTOF/MS-based metabolomic profiling. The two official pharmacopoeial species, *A. ebracteatus* and *A. ilicifolius*, were compared with the non-pharmacopoeial species *A. montanus* to evaluate species-level molecular discrimination and chemotaxonomic variation. ITS2 barcoding was used to confirm botanical identity, while LC–QTOF/MS analysis was employed to characterize species-specific chemical fingerprints and putative metabolite markers. This integrated molecular–chemical approach provides a comprehensive framework for distinguishing authentic Ngueak Pla Mo materials from non-authentic substitutes and supports quality control and standardization of this Thai medicinal crude drug.

## 2. Results

### 2.1. Botanical Authentication and Reference Sample Establishment of Acanthus Species

Botanical authentication was performed to establish reliable reference materials for the comparative authentication of *Acanthus* species associated with the Thai medicinal crude drug Ngueak Pla Mo. A total of 20 authenticated *Acanthus* samples were included in the ITS2-based molecular analysis, comprising seven samples of *A*. *ebracteatus* (NPM01–NPM07), five samples of *A. ilicifolius* (NPM08–NPM12), and eight samples of *A. montanus* (NPM13–NPM20). The two pharmacopoeial species, *A. ebracteatus* and *A. ilicifolius*, were used as official reference sources of Ngueak Pla Mo, whereas *A. montanus* was included as a morphologically related non-pharmacopoeial species for comparative authentication. The examined specimens were authenticated based on key macroscopic characters, including habit, leaf morphology, leaf margin, inflorescence structure, floral characteristics, and fruit morphology [34]. The pharmacopoeial species *A. ebracteatus* and *A. ilicifolius* shared several morphological similarities, particularly their spiny shrub habit and thick opposite leaves with dentate or spiny margins, reflecting their close taxonomic relationship and the practical difficulty of distinguishing these species in crude drug materials. However, species-level authentication was supported by diagnostic morphological features, especially floral and inflorescence characters. Representative morphological features and crude drug materials are shown in Figure 1. The authenticated samples were further established as reference materials for downstream molecular and chemical analyses. Fresh leaf materials were used for ITS2 DNA barcoding, while representative dried and powdered samples were prepared for LC–QTOF/MS-based chemical profiling. The inclusion of geographically diverse samples from different localities in Thailand provided a reference dataset for evaluating both intraspecific variation and interspecific differentiation among the studied taxa.

### 2.2. ITS2 Barcode Variation Reveals Multi-Layered Molecular Evidence for Differentiating Pharmacopoeial Acanthus Species from A. montanus

#### 2.2.1. Comparative ITS2 Nucleotide Variation and Candidate Diagnostic Sites Among *Acanthus* Species

Comparative analysis of the aligned ITS2 sequences revealed nucleotide variation among the examined *Acanthus* samples, resulting in eight haplotypes across three species: *A. ebracteatus*, *A. ilicifolius*, and *A. montanus* (Table S1). Overall, *A. ebracteatus* and *A. ilicifolius* exhibited relatively similar ITS2 sequence patterns, whereas *A. montanus* showed a clearly distinct nucleotide profile. The aligned sequence length also differed among species, with *A. ebracteatus* and *A. ilicifolius* showing lengths of approximately 228–230 bp, while *A. montanus* showed shorter aligned sequences of approximately 200–202 bp. This length reduction in *A. montanus* was mainly associated with several insertion/deletion regions in the aligned ITS2 matrix. However, although this difference may facilitate preliminary gel-based size discrimination, the relatively small difference between the amplicons may not be consistently resolved under routine electrophoretic conditions. Several nucleotide positions were identified as candidate diagnostic sites for distinguishing *A. montanus* from the two pharmacopoeial *Acanthus* species, *A. ebracteatus* and *A. ilicifolius*. In particular, the regions at positions 17–26, 41–54, and 153–161 showed prominent indel patterns in *A. montanus*, whereas these regions were largely conserved or present in *A. ebracteatus* and *A. ilicifolius*. In addition, fixed or nearly fixed nucleotide substitutions were observed at several positions, including 29, 32, 78, 107–108, 118, 147–149, 176–177, 188, 209, 217, 219, 225, 227–229, and 231. These sites collectively provide useful molecular characters for separating *A. montanus* from the *A. ebracteatus*–*A. ilicifolius* group. Although *A. ebracteatus* and *A. ilicifolius* could be separated into different haplotypes, their ITS2 sequences shared a high degree of similarity. Some nucleotide variations were observed among haplotypes within these two species, including substitutions and minor indels at positions such as 101, 107–108, 130, 142, 167, 169–170, and 176–177. However, these variable sites were not fully species-specific and may reflect intraspecific variation, shared ancestral polymorphism, or limited sequence divergence between the two closely related taxa. Therefore, while the ITS2 region appears informative for distinguishing *A. montanus* from the official Ngueak Pla Mo species, it may have limited discriminatory power for clearly separating *A. ebracteatus* from *A. ilicifolius* based solely on nucleotide variation. Collectively, the ITS2 nucleotide variation pattern supports the genetic distinctiveness of *A. montanus* from *A. ebracteatus* and *A. ilicifolius*. The candidate diagnostic regions identified in Table S1, particularly the indel-rich regions and species-associated nucleotide substitutions, may be useful for molecular authentication and for detecting potential substitution or misidentification of *Acanthus* crude drugs in herbal materials.

#### 2.2.2. Pairwise Genetic Distance and Barcode Gap Assessment

Pairwise genetic distance analysis of the ITS2 region was performed to evaluate the discriminatory potential of this marker for distinguishing *A. montanus* from the pharmacopoeial *Acanthus* species, *A. ebracteatus* and *A. ilicifolius*. The intraspecific genetic distances were relatively low within each species, with mean values of 0.0131 ± 0.01 for *A. ebracteatus*, 0.0322 ± 0.01 for *A. ilicifolius*, and 0.0010 ± 0.01 for *A. montanus* (Table 1). Among these taxa, *A. ilicifolius* showed the highest intraspecific variation, with a maximum distance of 0.0555, whereas *A. montanus* exhibited the lowest mean intraspecific distance, indicating limited sequence variation among its samples. Interspecific genetic distances between *A. montanus* and the two pharmacopoeial species were markedly higher than the observed intraspecific distances. The mean interspecific distance between *A. ebracteatus* and *A. montanus* was 0.1140 ± 0.03, while that between *A. ilicifolius* and *A. montanus* was 0.1150 ± 0.11 (Table 1). These values were substantially greater than the maximum intraspecific distances recorded for the compared taxa, indicating clear genetic divergence of *A. montanus* from both pharmacopoeial species based on the ITS2 region. Barcode gap assessment further supported the discriminatory capacity of ITS2 for separating *A. montanus* from *A. ebracteatus* and *A. ilicifolius*. The barcode gap was positive for both comparisons, with values of 0.0791 for *A. ebracteatus* vs. *A. montanus* and 0.0595 for *A. ilicifolius* vs. *A. montanus* (Table 1). These positive barcode gap values indicate that the interspecific divergence between *A. montanus* and the pharmacopoeial species exceeded the maximum intraspecific variation within the compared taxa. Therefore, the ITS2 region provides sufficient genetic resolution to distinguish *A. montanus* from the official pharmacopoeial sources of Ngueak Pla Mo, supporting its utility for detecting potential substitution or misidentification in *Acanthus* crude drug materials.

#### 2.2.3. ITS2 Haplotype Network Analysis

**Figure 2:**
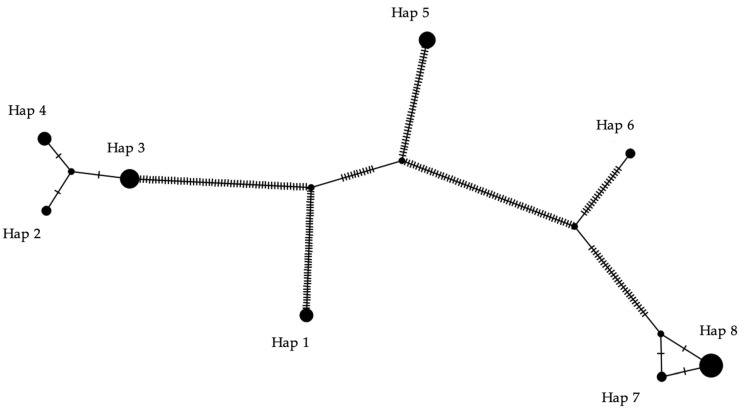
TCS haplotype network based on ITS2 sequences of *A. ebracteatus*, *A. ilicifolius*, and *A. montanus*. Each black circle represents a unique ITS2 haplotype. Hap 1–Hap 3 correspond to *A. ebracteatus*, Hap 4–Hap 5 correspond to *A. ilicifolius*, and Hap 6–Hap 8 correspond to *A. montanus*. Connecting lines indicate mutational relationships among haplotypes, while short hatch marks along the branches represent mutational steps. Small intermediate nodes indicate inferred unsampled or missing haplotypes. The clear separation of Hap 6–Hap 8 from Hap 1–Hap 5 supports the distinct ITS2 haplotype structure of *A. montanus* compared with the pharmacopoeial species *A. ebracteatus* and *A. ilicifolius*.

The TCS haplotype network based on ITS2 sequences identified eight haplotypes among the studied *Acanthus* samples (Figure 2). Hap 1–Hap 3 corresponded to *A. ebracteatus*, Hap 4–Hap 5 corresponded to *A. ilicifolius*, and Hap 6–Hap 8 corresponded to *A. montanus*. The network revealed distinct haplotype structuring among the three species, with *A. montanus* haplotypes clearly separated from those of the two pharmacopoeial species. Hap 6–Hap 8, representing *A. montanus*, formed a distinct haplotype group and were separated from Hap 1–Hap 5 by numerous mutational steps. This pattern indicates substantial ITS2 sequence divergence between *A. montanus* and the pharmacopoeial species (*A. ebracteatus* and *A. ilicifolius*). In contrast, Hap 1–Hap 3 of *A. ebracteatus* and Hap 4–Hap 5 of *A. ilicifolius* were positioned closer to each other within the network, reflecting a lower level of ITS2 divergence between the two pharmacopoeial taxa. Overall, the TCS haplotype network supports the discriminatory potential of ITS2 for distinguishing *A. montanus* from the official pharmacopoeial sources of Ngueak Pla Mo. This haplotype pattern is consistent with the pairwise genetic distance and barcode gap analyses, which demonstrated higher interspecific divergence between *A. montanus* and the two pharmacopoeial species than the intraspecific variation observed within each species.

#### 2.2.4. Mapping of Diagnostic SNPs onto Predicted ITS2 Secondary Structure

**Figure 3:**
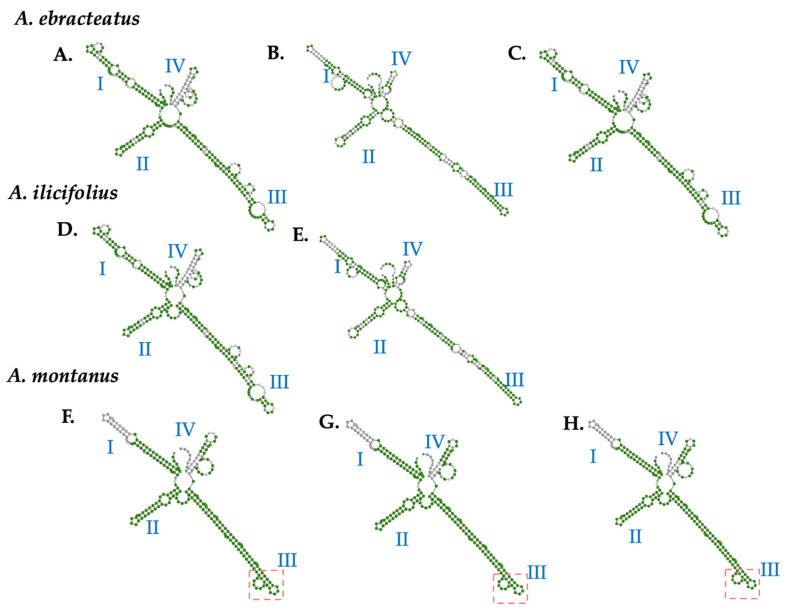
Predicted ITS2 secondary structures of eight haplotypes of *Acanthus ebracteatus*, *A. ilicifolius*, and *A. montanus*. The ITS2 secondary structures were predicted for haplotypes of the three studied *Acanthus* species: (**A**) *A. ebracteatus* Hap 1, (**B**) *A. ebracteatus* Hap 2, (**C**) *A. ebracteatus* Hap 3, (**D**) *A. ilicifolius* Hap 4, (**E**) *A. ilicifolius* Hap 5, (**F**) *A. montanus* Hap 6, (**G**) *A. montanus* Hap 7, and (**H**) *A. montanus* Hap 8. Roman numerals I–IV indicate the four conserved ITS2 helices. The predicted structures show generally similar four-helical organization among all haplotypes, while structural variation in helix regions and terminal loop patterns provides complementary evidence for distinguishing *A. montanus* from the pharmacopoeial species *A. ebracteatus* and *A. ilicifolius*. Orange dashed boxes highlight the distinctive terminal-loop configuration of helix III in *A. montanus*, which represents the most readily recognizable structural feature for differentiating this species from the two pharmacopoeial taxa.

The predicted ITS2 secondary structures of the eight *Acanthus* haplotypes exhibited the typical four-helical ITS2 organization, with helices I–IV recognizable in all haplotypes (Figure 3). Hap 1–Hap 3 of *A. ebracteatus* and Hap 4–Hap 5 of *A. ilicifolius* showed broadly comparable folding configurations, particularly in the central core region and the relative orientation of helices I, II, III, and IV. These similarities were consistent with the close ITS2 sequence relationship observed between the two pharmacopoeial species. However, minor structural variation was observed among haplotypes, especially in loop regions and terminal portions of the helices. In contrast, Hap 6–Hap 8 of *A. montanus* displayed a more distinct secondary structural profile. The most apparent differences were located in the terminal region of helix I, the configuration and extension of the long helix III, and the loop organization around the central junction and helix IV. These structural differences corresponded to the diagnostic nucleotide substitutions and indel-rich regions detected in the ITS2 alignment, suggesting that sequence divergence in *A. montanus* was reflected not only at the nucleotide level but also in the predicted ITS2 folding pattern.

The helix transfer similarity values further supported the structural differentiation among the haplotypes (Table 2). In *A. ebracteatus*, Hap 1 and Hap 3 showed identical and high transfer similarity values for helices I–III, with values of 95.238, 91.667, and 93.750, respectively, whereas helix IV showed no transfer similarity. Hap 2 displayed reduced similarity values for helices I–III and a moderate value for helix IV, indicating some intraspecific structural variation. A comparable pattern was observed in *A. ilicifolius*, in which Hap 4 showed relatively high transfer similarity for helices I–III, while Hap 5 showed lower values in helices I and III and a moderate value for helix IV. By contrast, Hap 6–Hap 8 of *A. montanus* consistently showed lower similarity in helix I but complete transfer similarity in helices II and III, with helix IV values ranging from 30.000 to 40.000. This species-associated helix transfer pattern indicates that *A. montanus* possesses a distinct ITS2 secondary structural profile compared with *A. ebracteatus* and *A. ilicifolius*. Together, the predicted structural models and helix transfer similarity values provide complementary evidence supporting the separation of *A. montanus* from the official pharmacopoeial sources of Ngueak Pla Mo.

#### 2.2.5. ITS2-Based Phylogenetic Analysis

**Figure 4:**
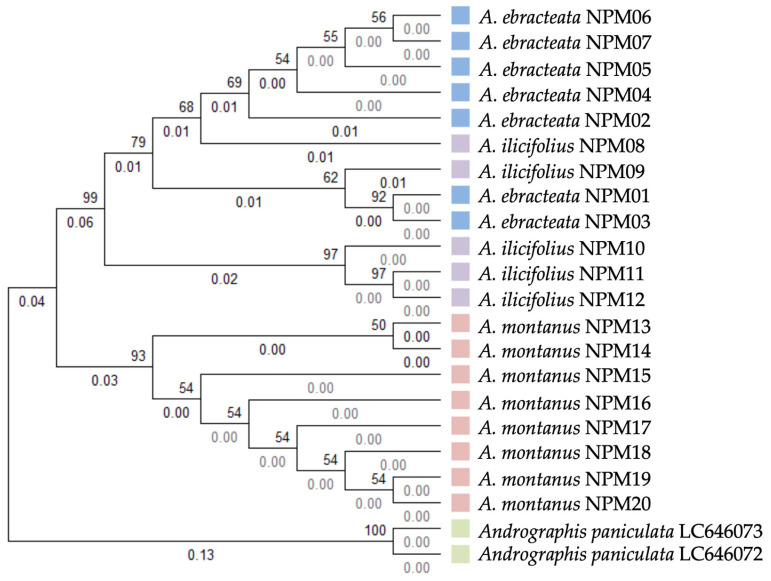
Neighbor-Joining phylogenetic tree and genetic divergence based on ITS2 sequences of *Acanthus ebracteatus*, *A. ilicifolius*, and *A. montanus*. The analysis included 20 ITS2 sequences of *Acanthus* samples: seven samples of *A. ebracteatus* (NPM01–NPM07), five samples of *A. ilicifolius* (NPM08–NPM12), and eight samples of *A. montanus* (NPM13–NPM20). Two ITS2 sequences of *Andrographis paniculata* (LC646072 and LC646073) were included as outgroups. Bootstrap values are shown above the branches, and branch lengths indicate genetic distance. Colored squares indicate species identity: blue, *A. ebracteatus*; purple, *A. ilicifolius*; pink, *A. montanus*; and green, *A. paniculata*.

The Neighbor-Joining (NJ) phylogenetic tree based on ITS2 sequences separated the studied *Acanthus* samples from the outgroup *Andrographis paniculata* (LC646072 and LC646073) (Figure 4). The two *A. paniculata* sequences clustered together with strong bootstrap support and were clearly positioned outside the *Acanthus* lineage, confirming their suitability as outgroups. Within the ingroup, all *Acanthus* accessions formed a distinct lineage, with the two pharmacopoeial species, *A. ebracteatus* and *A. ilicifolius*, generally grouped separately from *A. montanus*. The branch separating the *A. montanus* cluster from the *A. ebracteatus*–*A. ilicifolius* group showed a relatively longer genetic distance, indicating greater ITS2 divergence between *A. montanus* and the official Ngueak Pla Mo species. The *A. montanus* accessions (NPM13–NPM20) formed a distinct cluster with high bootstrap support, supporting the phylogenetic separation of this non-pharmacopoeial species from the two pharmacopoeial *Acanthus* species. Most *A. montanus* samples exhibited very short terminal branch lengths, suggesting low ITS2 variation within this species, consistent with the low intraspecific genetic distance observed in the pairwise distance analysis. In contrast, *A. ebracteatus* and *A. ilicifolius* were not resolved as completely independent monophyletic groups. Several *A. ilicifolius* accessions were positioned within or close to the *A. ebracteatus* lineage, particularly NPM08 and NPM09, whereas *A. ilicifolius* NPM10–NPM12 formed a separate subcluster with high bootstrap support. Similarly, *A. ebracteatus* samples formed several closely related subclusters, but their relationships overlapped with some *A. ilicifolius* accessions. This topology indicates close genetic relatedness and limited ITS2 resolution for clearly distinguishing *A. ebracteatus* from *A. ilicifolius*. Overall, the NJ phylogenetic analysis supports the discriminatory utility of ITS2 for separating *A. montanus* from the official pharmacopoeial sources of Ngueak Pla Mo. However, the intermingled placement and short genetic distances between *A. ebracteatus* and *A. ilicifolius* indicate that ITS2 alone may be insufficient for reliable species-level discrimination between these two pharmacopoeial taxa. These phylogenetic results are consistent with the pairwise genetic distance, barcode gap, haplotype network, and ITS2 secondary structure analyses, all of which support the clear molecular differentiation of *A. montanus* from the pharmacopoeial *Acanthus* species.

### 2.3. LC–QTOF/MS-Based Species Discrimination and Candidate Marker Prioritization

#### 2.3.1. Metabolite Annotation and Ontology Classification

**Figure 5:**
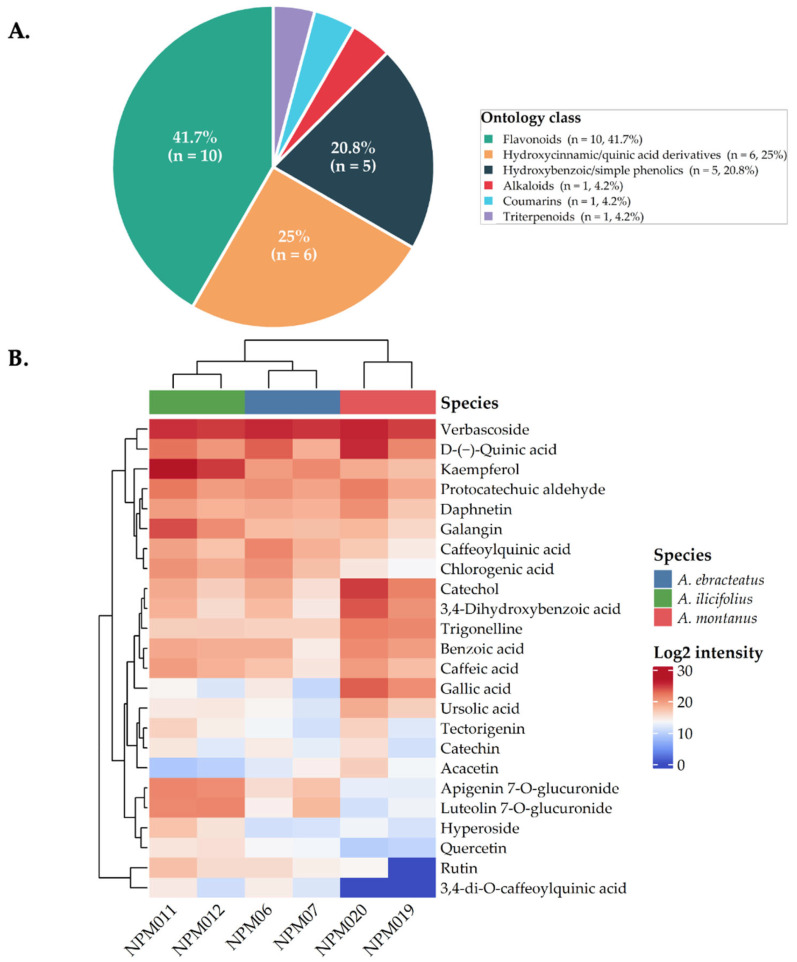
Metabolite profiling of *Acanthus* accessions. (**A**) Ontology distribution of the 24 annotated metabolites. (**B**) Hierarchical clustering heatmap showing species-specific metabolite patterns across six accessions.

Untargeted LC–QTOF/MS profiling, followed by MS-DIAL processing and multi-library spectral matching, yielded 24 annotated metabolites that met the predefined criteria of mass error ≤±5.0 ppm and identification score >1.0 across the six *Acanthus* accessions. These metabolites were assigned to six ontology classes (Figure 5A). Flavonoids represented the largest class (*n* = 10, 41.7%), followed by hydroxycinnamic/quinic acid derivatives (*n* = 6, 25.0%) and hydroxybenzoic acids/simple phenolics (*n* = 5, 20.8%). Alkaloids, coumarins, and triterpenoids were each represented by a single compound (*n* = 1, 4.2%), corresponding to trigonelline, daphnetin, and ursolic acid, respectively. Overall, phenolic-related compounds accounted for 87.5% of the annotated metabolites, indicating the phenolic-rich chemical profile of *Acanthus* species.

#### 2.3.2. Species-Specific Metabolite Patterns Revealed by Hierarchical Clustering

Hierarchical clustering of samples and metabolites, visualized as a heatmap (Figure 5B), revealed that the six accessions clustered according to species, indicating that the annotated metabolite panel reflected species-level chemical differences. Three tentative chemotypes were apparent. *A. ilicifolius* (NPM011 and NPM012) showed comparatively elevated levels of flavonoid glucuronides, particularly apigenin 7-*O*-glucuronide and luteolin 7-*O*-glucuronide, together with kaempferol and verbascoside. *A. ebracteatus* (NPM06 and NPM07) presented an intermediate profile, sharing several phenylpropanoids with *A. ilicifolius* at moderate intensities. *A. montanus* (NPM019 and NPM020) was characterized by higher relative abundances of hydroxybenzoic acids and simple phenolics, including catechol, 3,4-dihydroxybenzoic acid, gallic acid, benzoic acid, caffeic acid, and trigonelline, alongside the lowest levels of rutin and 3,4-di-*O*-caffeoylquinic acid among the three species.

#### 2.3.3. Unsupervised Prioritization by Principal Component Analysis (PCA)

**Figure 6:**
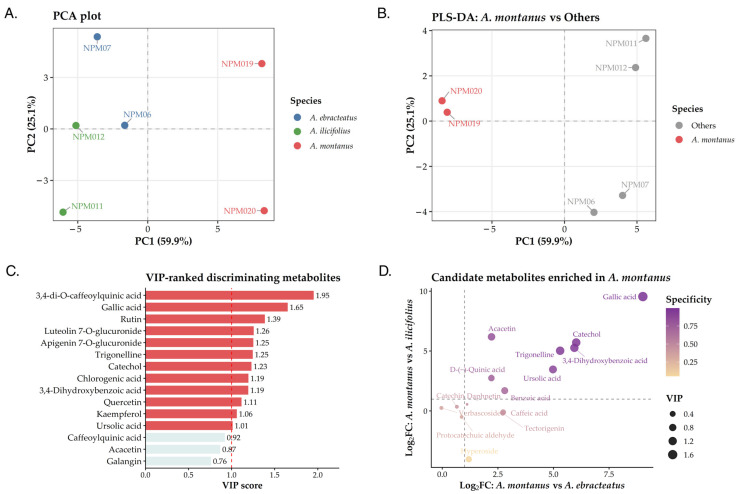
Multivariate analysis and marker prioritization for *A. montanus*. (**A**) PCA score plot showing species-level clustering. (**B**) PLS-DA score plot comparing *A. montanus* with the combined reference group (“Others”). (**C**) VIP-ranked discriminating metabolites; the dashed line indicates VIP = 1. (**D**) Bubble plot of *A. montanus*-enriched metabolites based on fold-changes, VIP score, and specificity.

To examine whether the species-level structure observed in the heatmap could be recovered without prior class labels, principal component analysis (PCA) was applied to the Pareto-scaled, log_2_-transformed metabolite intensities. The first two principal components accounted for 85.0% of the total variance (PC1: 59.9%; PC2: 25.1%) and arranged the six accessions into three species-consistent groupings (Figure 6A). PC1 primarily separated *A. montanus* from the two mangrove species, whereas PC2 further distinguished *A. ebracteatus* from *A. ilicifolius*.

#### 2.3.4. Exploratory Supervised Discrimination by PLS-DA

A supervised partial least squares discriminant analysis (PLS-DA) model was then constructed using *A. montanus* as the target class, while *A. ebracteatus* and *A. ilicifolius* were combined as the reference class. The model reproduced the main separation observed in PCA, distinguishing *A. montanus* from the reference class along component 1 (Figure 6B). LOOCV yielded a BER of 0 for both components across all three prediction distances (maximum, centroid, and Mahalanobis), indicating correct classification of all samples during cross-validation. Within the reference class, *A. ilicifolius* and *A. ebracteatus* also showed partial separation along component 2. Together with the PCA results, these findings support species-related chemical differences in the annotated metabolite panel. However, the results should be interpreted cautiously given the limited sample size.

#### 2.3.5. VIP-Based Identification of Discriminating Metabolites

VIP scores extracted from the PLS-DA model were used to rank metabolites according to their contribution to the discrimination of *A. montanus* from the two reference species (Figure 6C). Twelve metabolites exceeded the VIP > 1 threshold and were retained as candidate discriminators, including 3,4-di-*O*-caffeoylquinic acid, gallic acid, rutin, luteolin 7-*O*-glucuronide, apigenin 7-*O*-glucuronide, trigonelline, catechol, chlorogenic acid, 3,4-dihydroxybenzoic acid, quercetin, kaempferol, and ursolic acid. The highest VIP score was observed for 3,4-di-*O*-caffeoylquinic acid, indicating a strong contribution to interspecies discrimination. However, VIP score reflects discriminatory importance rather than direction of abundance change; therefore, additional fold-change and specificity analyses were required to identify metabolites that were positively enriched in *A. montanus*.

#### 2.3.6. Candidate Quality-Control Markers Based on Abundance and Specificity

To prioritize metabolites suitable for authentication and quality-control purposes, VIP-selected metabolites were further evaluated using pairwise log_2_ fold-change and specificity values (Figure 6D). This analysis distinguished metabolites that contributed to class separation from those that were specifically enriched in *A. montanus*. Gallic acid emerged as the strongest positive candidate marker, showing high enrichment relative to both *A. ebracteatus* and *A. ilicifolius*, together with the highest specificity score. Catechol, 3,4-dihydroxybenzoic acid, trigonelline, and ursolic acid also showed positive fold-changes and high specificity, supporting their use as complementary candidate authentication markers. In contrast, compounds such as 3,4-di-*O*-caffeoylquinic acid, rutin, and flavonoid glucuronides showed high VIP scores but were not preferentially enriched in *A. montanus*. These metabolites are therefore useful for explaining species discrimination but are less suitable as positive quality-control markers for *A. montanus*. Overall, gallic acid, catechol, 3,4-dihydroxybenzoic acid, trigonelline, and ursolic acid were prioritized as the most promising candidate markers for further validation.

Taken together, the stepwise workflow, encompassing ontology classification, hierarchical clustering, PCA, PLS-DA, and VIP-guided fold-change analysis, identified a concise panel of candidate markers for *A. montanus*. Gallic acid, catechol, 3,4-dihydroxybenzoic acid, trigonelline, and ursolic acid consistently distinguished *A. montanus* from *A. ilicifolius* and *A. ebracteatus* in this dataset. However, because each species was represented by only two accessions, these findings should be regarded as preliminary. Formal qualification of these metabolites as quality-control markers will require validation using independent and geographically diverse sample sets, together with the development of quantitative assays that establish limits of detection, limits of quantification, and stability profiles.

## 3. Discussion

### 3.1. ITS2-Based Molecular Authentication Reveals the Genetic Distinctiveness of Acanthus montanus from Pharmacopoeial Ngueak Pla Mo Species

Accurate botanical authentication is essential for medicinal plant quality control, particularly when closely related species share similar morphology, vernacular names, or traditional uses. Although macroscopic and microscopic pharmacognostic examinations remain important, their discriminatory power can be limited when crude drugs are dried, fragmented, powdered, or processed, because diagnostic morphological organs may be absent or degraded. DNA barcoding provides a complementary molecular approach for species identification and adulterant detection because it is less affected by plant developmental stage, environmental conditions, and chemical variability [27,35,36]. This approach has been increasingly applied to herbal medicine authentication and is particularly useful for distinguishing authentic medicinal plants from closely related substitutes or adulterants [27,37].

Among commonly used plant barcode regions, including *rbc*L, *mat*K, *psb*A–*trn*H, and ITS/ITS2, the nuclear ITS2 region has been widely recognized as an effective marker for medicinal plant identification because of its short length, high amplification efficiency, and relatively high interspecific variation [22,24]. Large-scale validation studies have shown that ITS2 performs well in identifying medicinal plants and closely related species, and it has been applied successfully to several medicinal plant groups, such as *Dendrobium* [38], *Panax* [39,40], *Zanthoxylum* [41,42], Selaginellaceae [43], and medicinal Acanthaceae [26]. In addition to primary sequence analysis, ITS2 secondary structure can provide further taxonomic information by mapping nucleotide substitutions and indels onto conserved helix and loop regions, thereby supporting species discrimination when sequence variation is structurally informative [24,44,45]. Previous studies on Thai medicinal Acanthaceae also support the utility of DNA barcoding approaches for authenticating medicinal plant materials, including *A. ebracteatus* [26,46].

In the present study, ITS2-based analyses consistently demonstrated the genetic distinctiveness of *A. montanus* from the pharmacopoeial Ngueak Pla Mo species, *A. ebracteatus* and *A. ilicifolius*. Comparative ITS2 sequence analysis identified prominent indel-rich regions and species-associated nucleotide substitutions in *A. montanus*, while pairwise genetic distance and barcode gap assessment showed that interspecific divergence between *A. montanus* and the pharmacopoeial species exceeded the maximum intraspecific variation. This finding is consistent with the fundamental criterion of DNA barcoding, in which effective species discrimination is supported when interspecific divergence is greater than intraspecific variation [22,24,27]. The clear molecular separation of *A. montanus* also agrees with previous studies showing that ITS2 is particularly informative for distinguishing closely related medicinal taxa and detecting non-authentic substitutes in herbal materials [24,38,43]. In medicinal Acanthaceae, DNA barcoding and ITS2-based approaches have likewise been reported to provide useful resolution for authenticating species used in Thai traditional medicine, supporting the relevance of this marker for the quality control of Acanthaceae crude drugs [26,46]. The TCS haplotype network and NJ phylogenetic tree further supported the separation of *A. montanus*, as Hap 6–Hap 8 and accessions NPM13–NPM20 formed a distinct group apart from the *A. ebracteatus*–*A. ilicifolius* lineage. This pattern indicates that the ITS2 variation observed in *A. montanus* was not limited to isolated nucleotide differences but was consistently reflected across multiple analytical layers, including haplotype structure, phylogenetic topology, genetic distance, and barcode gap assessment. Because ITS2 contributes to pre-rRNA processing and ribosome biogenesis, its secondary structure is subject to functional constraints, although sequence variation may accumulate in less-conserved helices and loop regions provided that the overall folding pattern is maintained [47,48]. Consistent with this, the predicted ITS2 structures of *A. montanus* showed distinct configurations, particularly in helices I and III and in several loop regions. These features may reflect lineage-specific accumulation of substitutions and indels during evolutionary divergence and may therefore provide complementary taxonomic signals. Nevertheless, because the structures were computationally predicted, their direct functional significance remains to be experimentally confirmed. The incorporation of ITS2 secondary structure can strengthen taxonomic interpretation by allowing substitutions and indels to be evaluated in relation to conserved structural motifs, especially when primary sequence variation alone provides insufficient resolution among closely related taxa [24,44,45]. Accordingly, the structural differentiation observed in *A. montanus* provides additional evidence of its molecular distinctiveness from the pharmacopoeial Ngueak Pla Mo species. In contrast, *A. ebracteatus* and *A. ilicifolius* showed closer ITS2 relationships, shorter genetic distances, and partial phylogenetic overlap, indicating limited ITS2 resolution for separating these two pharmacopoeial taxa. Similar limitations have been reported in other medicinal plant groups, where ITS2 can effectively distinguish non-authentic or more divergent taxa but may show reduced resolution among very closely related species because of recent divergence, shared ancestral polymorphism, or insufficient barcode variation [24,27,37]. The low interspecific divergence observed between *A. ebracteatus* and *A. ilicifolius* indicates that ITS2 alone does not provide sufficient diagnostic variation for consistent species-level discrimination. Therefore, additional loci may be required to improve resolution; for example, the highly variable chloroplast *ycf*1 [49] or *psb*A-*trn*H [21,50] region could be evaluated as a complementary marker alongside ITS2, although its discriminatory performance should first be validated using broader population-level sampling of both species. Thus, while ITS2 provides robust molecular evidence for detecting *A. montanus* as a non-pharmacopoeial substitute, it should be interpreted together with pharmacognostic characters and metabolite profiling for comprehensive Ngueak Pla Mo authentication. This integrated approach is particularly important for crude drug quality control, where accurate species identity and chemical consistency are both required to ensure authenticity, safety, and therapeutic reliability.

### 3.2. LC–QTOF/MS-Based Chemical Profiling Provides Candidate Quality-Control Markers

This study applied an untargeted LC–QTOF/MS metabolomics workflow to differentiate *A. montanus* from two related species with similar morphology, *A. ilicifolius* and *A. ebracteatus*, and to identify candidate chemical markers suitable for authentication and quality control. The annotated metabolite panel was dominated by phenolic compounds, consistent with previous reports describing the genus *Acanthus* as a rich source of phenylpropanoid glycosides, phenylethanoids, flavonoids, triterpenoids, hydroxybenzoic acids, and simple phenols [5]. In the Thai Herbal Pharmacopoeia, both Ngueak Pla Mo Dok Khao (*A. ebracteatus*) and Ngueak Pla Mo Dok Muang (*A. ilicifolius*) are chemically identified using the same reference marker, verbascoside/acteoside, reflecting the shared occurrence of phenylethanoid glycosides in the two pharmacopoeial species [2,3]. However, the LC–QTOF/MS results of the present study showed that this compound or related phenylethanoid-type metabolites were also detected in *A. montanus*. This indicates that the pharmacopoeial marker is useful for confirming the presence of characteristic *Acanthus*-type phenolic constituents but may not be sufficiently species-specific for distinguishing pharmacopoeial Ngueak Pla Mo materials from non-pharmacopoeial substitutes. Therefore, metabolomics-based marker discovery was required to identify additional differential chemical markers capable of separating *A. montanus* from *A. ebracteatus* and *A. ilicifolius*.

Hierarchical clustering and unsupervised PCA independently recovered species-consistent groupings, suggesting that the observed chemical differences reflected species-related variation rather than supervised classification effects. *A. ilicifolius* and *A. ebracteatus* clustered more closely with each other than with *A. montanus*, consistent with plastome evidence of high sequence conservation and recent differentiation within the mangrove *Acanthus* group [51]. The broader separation of *A. montanus* aligns with the reported divergence between mangrove and non-mangrove *Acanthus* relatives and with the overlapping, yet distinct phytochemical profiles reported across these species [5,51], supporting the value of metabolite-based authentication for closely related *Acanthus* species. The enrichment of flavonoid glucuronides in both mangrove species likely reflects shared flavonoid-related biosynthetic capacity, consistent with transcriptome–metabolome evidence of active flavonoid biosynthesis in *A. ilicifolius* [52] and the conserved genomic background of the mangrove *Acanthus* group [51]. In contrast, the hydroxybenzoic acid/simple phenolic profile observed in *A. montanus* aligns with previous reviews describing phenolic compounds as important constituents across the genus *Acanthus* [5]. The finding that some metabolites contributed strongly to class separation without being preferentially enriched in *A. montanus* highlights a key limitation of relying on VIP scores alone. Combining discriminatory importance with enrichment direction and specificity therefore provides a more practical basis for authentication marker selection. This strategy is consistent with the growing application of untargeted metabolomics and chemometrics for distinguishing closely related or easily confused medicinal plants. LC–QTOF/MS profiling combined with multivariate analysis has been demonstrated as an effective approach for species authentication and chemical marker identification, as shown in *Panax* species [53] and *Fritillariae* Bulbus [54]. The present study extends this approach to the genus *Acanthus*, which is relevant because *A. montanus*, *A. ilicifolius*, and *A. ebracteatus* are among the most commonly used species in traditional medicine. From a practical perspective, the identified markers may support the authentication of *A. montanus* raw materials and the verification of multi-species traditional preparations containing *Acanthus*. Beyond their statistical contribution to species discrimination, the biosynthetic origins and reported distribution of these metabolites within the genus provide additional support for their potential relevance as chemical markers. The chemical characteristics of the metabolites provide biological plausibility for the observed discrimination. Gallic acid and 3,4-dihydroxybenzoic acid, also known as protocatechuic acid, are hydroxybenzoic acids associated with shikimate- and phenylpropanoid-related phenolic metabolism, consistent with the reported occurrence of phenolic acids, phenylethanoid glycosides, and other aromatic phenolic constituents in the genus *Acanthus* [1,5,55,56]. In particular, acanmontanoside, a phenylethanoid glycoside containing a syringic acid moiety, has been isolated from *A. montanus* [55], whereas phenylethanoid and hydroxycinnamic acid glycosides have been reported from *A. ilicifolius* [1,56]. Catechol, another simple aromatic phenolic compound, may reflect species-dependent differences in the formation or turnover of related phenolic metabolites, although its exact biosynthetic origin cannot be determined from the present untargeted metabolomics data. Ursolic acid is a pentacyclic triterpenoid biosynthesized through the isoprenoid pathway via cyclization of 2,3-oxidosqualene to α-amyrin followed by CYP716A-mediated oxidation [57,58]. The previous identification of ursolic acid, α-amyrin, and related triterpenoids in *Acanthus* species is therefore consistent with the occurrence of this marker in the present dataset [5,59]. Trigonelline is a pyridine alkaloid formed through the N-methylation of nicotinic acid by nicotinate N-methyltransferase [60,61]; however, its occurrence and biosynthetic significance in *Acanthus* remain insufficiently characterized and require further confirmation. Collectively, these relationships indicate that the proposed marker panel is broadly consistent with known phenolic, triterpenoid, and pyridine-related metabolism in plants. Nevertheless, these pathway associations should not be interpreted as direct evidence of differential pathway activity, which would require additional transcriptomic, enzymatic, or targeted metabolic investigations.

Reliance on a single compound is generally insufficient, as secondary metabolite levels may vary with genetic, environmental, and processing-related factors. Monitoring multiple complementary markers is therefore more consistent with established quality-control approaches for herbal medicines [30]. Several proposed markers are available as reference standards and may be monitored using routine chromatographic methods, supporting their potential development into targeted quality-control assays. Among the detected metabolites, gallic acid and rutin appear to be promising candidates for future targeted quantification using HPLC or LC–MS/MS, particularly because certified reference standards are commercially available. Gallic acid may be evaluated as a positively enriched marker for *A. montanus*, whereas rutin may provide complementary discriminatory value based on its differential abundance among the examined species. However, their applicability to routine quality control will require the development and full validation of targeted analytical methods. Several limitations should be noted. Each species was represented by only two accessions, limiting statistical power and potentially overstating the separation observed in PCA and PLS-DA. The proposed markers, including the strongly enriched gallic acid, should therefore be regarded as preliminary. Future studies should validate these candidates using larger and geographically diverse sample sets, followed by development of quantitative assays with established limits of detection, limits of quantification, and stability profiles.

### 3.3. Integrating Molecular and Chemical Evidence for Robust Authentication and Quality Control of Ngueak Pla Mo Crude Drugs

Although ITS2 barcoding provided strong molecular evidence for distinguishing *A. montanus* from the pharmacopoeial Ngueak Pla Mo species, DNA-based authentication alone does not fully address all aspects of crude drug quality control. DNA barcoding is highly effective for confirming botanical origin and detecting substitution or misidentification, but its application can be limited in extensively dried, powdered, processed, extracted, or multi-ingredient herbal products, where DNA may be degraded, fragmented, or absent. Moreover, DNA barcoding cannot directly evaluate chemical composition, bioactive marker abundance, or batch-to-batch metabolite consistency. Therefore, an integrated or orthogonal strategy combining molecular identification with chemical and pharmacognostic analyses has been increasingly recommended for herbal quality assurance [36,37]. This integrated strategy has been increasingly applied in medicinal plant quality control. Sánchez et al. combined DNA barcoding with UHPLC–MS analysis to authenticate commercial medicinal plant samples, including valerian, linden, tea, and chamomile, demonstrating that molecular identification and metabolite profiling provide complementary information on botanical identity and chemical quality [62]. In another example, metabolomics coupled with DNA barcoding was used to identify discriminatory quality markers for *Cimicifuga* species, supporting the value of combining taxonomic confirmation with chemical marker discovery [63]. A similar integrated approach was also applied in our previous kratom study, where DNA barcoding was combined with UHPLC, GC–MS, and LC–QTOF/MS chemical profiling to authenticate *Mitragyna speciosa* variants and discriminate them from closely allied species for kratom material quality control [64]. These studies collectively show that DNA barcoding answers the question of “what species is present,” whereas chemical profiling addresses “what metabolites are present and whether the material is chemically consistent”. In the present study, this molecular–chemical framework is particularly relevant for Ngueak Pla Mo because the crude drug name refers to more than one pharmacopoeial *Acanthus* species and may be confused with morphologically related non-pharmacopoeial taxa. ITS2 analysis established the genetic distinctiveness of *A. montanus* from *A. ebracteatus* and *A. ilicifolius*, while LC–QTOF/MS profiling provides an additional layer of evidence by characterizing metabolite variation and candidate chemical markers among the studied materials. This combined approach is stronger than either method alone: ITS2 confirms species identity and detects substitution, whereas LC–QTOF/MS evaluates phytochemical composition, marker abundance, and chemical consistency relevant to crude drug quality. Therefore, integrating molecular authentication with metabolite profiling provides a more robust strategy for distinguishing authentic Ngueak Pla Mo materials from non-pharmacopoeial substitutes and supports future standardization, regulatory quality control, and pharmacopoeial authentication of *Acanthus* crude drugs. In the future, the diagnostic ITS2 substitutions and indels identified for *A. montanus* could be developed into rapid species-specific assays, including PCR, qPCR, or colorimetric LAMP (loop-mediated isothermal amplification). Combined with simplified DNA extraction, LAMP may be particularly suitable for field use because it requires minimal equipment and permits visual interpretation. The proposed chemical markers could provide complementary confirmation through portable HPTLC or targeted screening. However, assay specificity, sensitivity, reproducibility, and performance across diverse accessions, processed materials, and multispecies preparations must be validated before regulatory implementation.

## 4. Materials and Methods

### 4.1. Plant Materials, Voucher Specimens, and Botanical Authentication

**Figure 7:**
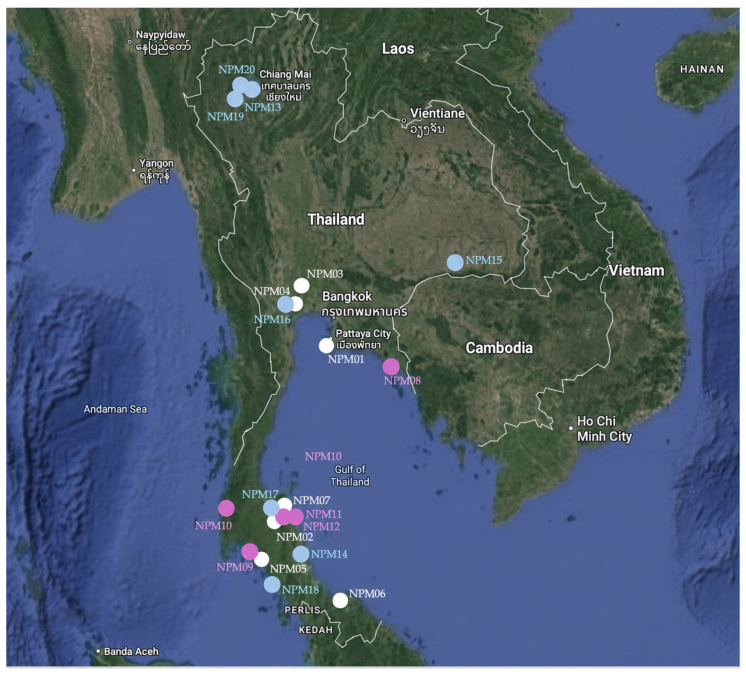
Geographic distribution of sampling sites for the 20 authenticated accessions of *A. ebracteatus*, *A. ilicifolius*, and *A. montanus* collected across Thailand. Different colors indicate the three studied species, and sample codes correspond to those listed in Table 3. The base map was adapted from Google Maps (accessed on 26 July 2026).

Authenticated leaf materials of *Acanthus* species associated with the Thai medicinal crude drug “Ngueak Pla Mo” were used in this study. The two pharmacopoeial species, *A. ebracteatus* Vahl and *A. ilicifolius* L., were included as official botanical sources, while *A. montanus* L. was selected as a morphologically related non-pharmacopoeial species for comparative authentication. This sample design enabled the evaluation of both molecular and chemical evidence for distinguishing official Ngueak Pla Mo species from a closely related non-official *Acanthus* species. All plant materials were taxonomically authenticated by Miss Wannaree Charoensup, a botanist at the Faculty of Pharmacy, Chiang Mai University, Chiang Mai, Thailand. Species identification was conducted based on key morphological traits, including leaf shape and margin, floral morphology, and fruit characteristics. The authenticated specimens were further compared with taxonomic descriptions in the Thai Herbal Pharmacopoeia and the Plant Genera of Thailand database to ensure consistency with accepted diagnostic characters. For ITS2 DNA barcoding analysis, fresh leaves of *A. ebracteatus*, *A. ilicifolius*, and *A. montanus* were collected from different geographical locations in Thailand (Table 3, Figure 7). Fully developed, healthy leaves without visible lesions, pathogen infection, or mechanical damage were selected for DNA analysis. The leaf surfaces were cleaned with 70% ethanol, and the samples were immediately placed in zip-lock plastic bags containing silica gel for rapid desiccation. The dried leaf materials were maintained under moisture-free conditions until genomic DNA extraction. For chemical profiling and marker confirmation, representative samples of each species were prepared for LC–QTOF/MS analyses. *A. ebracteatus* was collected from Mueang Songkhla District, Songkhla Province (NPM01), and Pho Sadet District, Nakhon Si Thammarat Province (NPM02). *A. ilicifolius* was collected from Pho Sadet District, Nakhon Si Thammarat Province (NPM03), and Mueang Nakhon Si Thammarat District, Nakhon Si Thammarat Province (NPM04). *A. montanus* was collected from the Faculty of Pharmacy, Chiang Mai University, Mueang Chiang Mai District, Chiang Mai Province (NPM05), and Queen Sirikit Botanic Garden, Mae Rim District, Chiang Mai Province (NPM06). The collected plant materials were dried in a hot-air oven at 50 °C for 24 h, pulverized into fine powder, and stored in airtight containers under desiccated conditions until chemical analysis.

### 4.2. Molecular Authentication Using ITS2 DNA Barcoding

#### 4.2.1. Genomic DNA Extraction

Leaf samples from the three *Acanthus* species, namely *A. ebracteatus*, *A. ilicifolius*, and *A. montanus*, were pulverized into a fine powder using a mortar and pestle under liquid nitrogen to minimize DNA degradation. Genomic DNA was extracted from approximately 100 mg of powdered leaf material using the DNeasy Plant Mini Kit (Qiagen, Hilden, Germany), according to the manufacturer’s instructions. In brief, the powdered tissue was lysed using the kit lysis buffer, and the lysate was clarified by centrifugation to remove cellular debris. The supernatant was transferred to a silica membrane spin column, where DNA was selectively bound, washed to eliminate residual contaminants and potential PCR inhibitors, and finally eluted in the kit elution buffer. The extracted DNA was stored at −20 °C prior to PCR amplification.

#### 4.2.2. PCR Amplification and Sanger Sequencing of the ITS2 Region

PCR amplification of the ITS2 region was performed using a T960 PCR thermocycler (Drawell, Chongqing, China) with the primer pair S2F (5′-ATGCGATACTTGTGTGAAT-3′) and S3R (5′-GACGCTTCTCCAGACTACAAT-3′). Each PCR mixture consisted of 100–200 ng of genomic DNA, 2× PCR Buffer for KOD FX Neo, nuclease-free water, KOD FX Neo DNA polymerase (TOYOBO Life Science, Osaka, Japan), and forward and reverse primers. KOD FX Neo DNA polymerase was used according to the manufacturer’s instructions because of its high fidelity and robust amplification performance, particularly for plant DNA templates that may contain PCR-inhibitory compounds. The PCR conditions were as follows: initial denaturation at 94 °C for 2 min; 30 cycles of 94 °C for 15 s, 55 °C for 15 s, and 58 °C for 45 s; followed by a final extension at 65 °C for 5 min. Amplification success was verified by electrophoresis on 1.8% (*w*/*v*) agarose gels stained with RedSafe™ nucleic acid staining solution (iNtRON Biotechnology, Seongnam, Republic of Korea), and the bands were visualized under UV light using a Gel Documentation EZ Imager (Bio-Rad, Hercules, CA, USA). PCR products with clear target bands were purified and sequenced in both directions using an ABI PRISM 3730 XL Genetic Analyzer (Applied Biosystems, Waltham, MA, USA).

#### 4.2.3. ITS2 Sequence Analysis, Genetic Distance, Haplotype Network, and Secondary Structure Prediction

Raw Sanger chromatogram files were inspected, assembled, trimmed, and edited using BioEdit version 7.2.6. Low-quality regions at both sequence ends and ambiguous nucleotide positions were manually checked against the electropherograms. Forward and reverse reads were assembled into consensus sequences, and only high-quality ITS2 sequences were retained for downstream analyses. Multiple sequence alignment of ITS2 sequences was performed using ClustalW implemented in BioEdit version 7.2.6, followed by manual adjustment to ensure positional homology. The final alignment was used to characterize nucleotide variation among *A. ebracteatus*, *A. ilicifolius*, and *A. montanus*, with particular emphasis on variable sites, indels, and candidate diagnostic SNPs that could distinguish *A. montanus* from the two pharmacopoeial species. Pairwise genetic distances were estimated using MEGA version 12 under the Maximum Composite Likelihood model to evaluate ITS2 sequence divergence within and between the studied taxa. The analysis included nucleotide substitutions involving both transitions and transversions. Uniform rates among sites and homogeneous substitution patterns among lineages were assumed. Gaps and missing data were treated using the pairwise deletion option. Standard errors of the estimated distances were calculated using the bootstrap method with 10,000 replicates. Intraspecific genetic distances were calculated within each species, whereas interspecific genetic distances were estimated between *A. montanus* and each of the two pharmacopoeial species, namely *A. ebracteatus* and *A. ilicifolius*. The barcode gap analysis was specifically performed to assess whether the ITS2 region could discriminate *A. montanus* from these two official medicinal plant sources. A positive barcode gap was inferred when the interspecific distance between *A. montanus* and the pharmacopoeial species exceeded the maximum intraspecific distance observed among the compared taxa. This approach was used to determine the discriminatory potential of ITS2 for detecting possible substitution or misidentification of *A. montanus* in relation to pharmacopoeial *Acanthus* crude drugs.

Following multiple sequence alignment, ITS2 haplotypes were identified from the aligned sequences of *A. ebracteatus*, *A. ilicifolius*, and *A. montanus* using DnaSP version 6 [65]. Prior to haplotype analysis, the alignment was manually inspected to minimize the influence of ambiguous bases, missing data, and poorly aligned indel-rich regions. Positions containing undefined nucleotide states were excluded where necessary to reduce analytical bias, while consistent indel patterns were recorded separately as complementary diagnostic characters for species discrimination. The cleaned ITS2 alignment was imported into DnaSP6 to identify unique haplotypes and to assign haplotype patterns among the studied samples. Haplotypes were then grouped according to species identity, corresponding to *A. ebracteatus*, *A. ilicifolius*, and *A. montanus*. The haplotype dataset was exported from DnaSP6 and subsequently used for network construction in PopART [66]. A TCS haplotype network was constructed using PopART software version 1.7 to visualize mutational relationships among ITS2 haplotypes and to assess species-level haplotype differentiation among the studied Acanthus taxa. In the network, each node represented a unique ITS2 haplotype, connecting branches indicated mutational steps between haplotypes, and small intermediate nodes represented inferred unsampled or missing haplotypes. This analysis was performed to determine whether *A. montanus* formed distinct ITS2 haplotypes separated from the pharmacopoeial species *A. ebracteatus* and *A. ilicifolius*.

For Prediction of ITS2 Secondary Structure, the accurate boundaries of the ITS2 region were determined using Hidden Markov Models. ITS2 secondary structures were predicted using homology modelling through the ITS2 Database Workbench [67], accessed on 18 April 2026, with a default threshold of 75% for helix transfer similarity. A gap opening penalty of 20 and a gap extension penalty of 4 were applied for transferred helices. Candidate diagnostic SNPs were subsequently mapped onto the predicted ITS2 secondary structures to determine whether the substitutions occurred in conserved helix or loop regions, thereby providing additional structural context for molecular discrimination among Acanthus species.

### 4.3. LC–QTOF/MS-Based Metabolite Profiling

#### 4.3.1. Plant Material and Sample Preparation

Six accessions from three *Acanthus* species were analysed: *A. ebracteatus* (NPM06, NPM07), *A. ilicifolius* (NPM011, NPM012), and *A. montanus* (NPM019, NPM020). Each accession represented a geographically distinct population, and mature plants were selected to reduce variation associated with developmental stage. Dried whole-plant material was used in accordance with the medicinal plant part specified in the Thai Herbal Pharmacopoeia. Approximately 500 g of the powdered plant material was subjected to Soxhlet extraction with 5.0 L of ethanol, corresponding to a solvent-to-material ratio of 10:1 (*v*/*w*). The extraction was carried out at approximately 60 °C for 20 h using a Soxhlet apparatus (Haberg, Germany). After extraction, the solvent was removed under reduced pressure with a rotary evaporator. The concentrated ethanolic extract was transferred to amber glass bottles and stored under refrigerated conditions until phytochemical analysis. The extract from each accession was thoroughly homogenized, and approximately 10 mg was weighed and extracted with 95% ethanol. The extracts were vortexed and centrifuged at 14,000 rpm for 10 min. The supernatants were collected, filtered through a syringe filter, and analyzed by LC–QTOF/MS. Given that two accessions per species were included, the metabolomics experiment was designed as an exploratory comparison of species-associated chemical profiles.

#### 4.3.2. LC–QTOF/MS Analysis

Untargeted metabolite profiling was performed using an Agilent 6545XT LC–QTOF/MS system (Agilent Technologies, CA, USA). Chromatographic separation was achieved on a Poroshell 120 EC-C18 column (2.1 × 100 mm, 2.7 µm) maintained at 50 °C, with an injection volume of 10 µL. The mobile phases consisted of water containing 0.1% formic acid (A) and acetonitrile containing 0.1% formic acid (B), delivered at a flow rate of 0.4 mL/min. A linear gradient was applied from 100% A to 100% B over 17 min, followed by column re-equilibration. Detection was performed using electrospray ionization (ESI) in both positive and negative ion modes. The source parameters were set as follows: capillary voltage, 4000 V; nebulizer pressure, 45 psi; drying gas temperature, 325 °C; and drying gas flow, 13 L/min. MS data were acquired over an *m*/*z* range of 40–1700, and MS/MS data were collected over an *m*/*z* range of 25–1000 using collision energies of 20 and 40 eV. Mass accuracy was maintained by continuous internal calibration using reference ions at *m*/*z* 121.0509 and 922.0098 in positive ion mode and *m*/*z* 112.9856 and 1033.9881 in negative ion mode.

#### 4.3.3. Data Processing and Metabolite Annotation

Raw LC–QTOF/MS data were processed in MS-DIAL v5.3 (RIKEN, Kanagawa, Japan) for peak picking, deconvolution, and alignment. Metabolite annotation was performed by matching precursor masses and MS/MS spectra against the MS-DIAL ESI (+/−), Fiehn/Vaniya Natural Products, and BMDMS-NP libraries. Annotations were accepted when the mass error was within ±5.0 ppm and the spectral identification score exceeded 1.0, yielding 24 confidently identified metabolites that were subsequently assigned to ontology classes based on chemical structure and biosynthetic origin.

#### 4.3.4. Multivariate Analysis and Identification of Candidate Authentication Markers

Log_2_-transformed intensities of the 24 identified metabolites were Pareto-scaled prior to multivariate analysis. All analyses were performed in R version 4.5.1. Principal component analysis (PCA) was performed to examine overall sample distribution and species clustering. Supervised partial least squares discriminant analysis (PLS-DA) was then performed using the mixOmics package [68], with *A. montanus* as the target class and *A. ilicifolius* and *A. ebracteatus* combined as the reference class for authentication purposes. Given the limited sample size, model performance was assessed using leave-one-out cross-validation (LOOCV). Classification performance was evaluated using the balanced error rate (BER), with predictions assessed using maximum, centroid, and Mahalanobis distance methods. Variable importance in projection (VIP) scores were calculated from the PLS-DA model, and metabolites with VIP > 1 were retained for marker prioritization. For each VIP-selected metabolite, pairwise log_2_ fold-changes were calculated for *A. montanus* relative to *A. ebracteatus* and *A. ilicifolius*. A relative specificity score ranging from 0 to 1 was calculated based on the relative enrichment of each metabolite in *A. montanus* across the three species, with higher values indicating greater preferential enrichment in *A. montanus*. Preliminary candidate markers were defined as metabolites meeting all three criteria: VIP > 1, log2FC ≥ 1 relative to each reference species, and a relative specificity score ≥0.60.

## 5. Conclusions

This study demonstrates that integrating ITS2 DNA barcoding with LC–QTOF/MS-based metabolite profiling provides complementary evidence for the authentication of Ngueak Pla Mo crude drugs. ITS2 consistently differentiated the non-pharmacopoeial species *A. montanus* from the official sources, *A. ebracteatus* and *A. ilicifolius*, through diagnostic nucleotide variation, positive barcode gaps, distinct haplotypes, phylogenetic separation, and predicted secondary-structure differences. However, its limited resolution between the two pharmacopoeial species indicates that additional loci may be required for their reliable discrimination. Chemical profiling revealed species-associated metabolite patterns and prioritized gallic acid, catechol, 3,4-dihydroxybenzoic acid, trigonelline, and ursolic acid as candidate markers for *A. montanus*. Although these markers remain preliminary because of the limited number of accessions, they provide a useful basis for future targeted validation. Overall, the integrated molecular–chemical approach offers a promising framework for pharmacopoeial authentication, routine quality control, and regulatory evaluation of *Acanthus* crude drugs. Future studies should evaluate additional barcode loci and broader population-level sampling to improve discrimination between *A. ebracteatus* and *A. ilicifolius*. Targeted HPLC or LC–MS/MS methods should also be developed to validate the proposed chemical markers and assess their suitability for routine quality-control applications.

## Figures and Tables

**Table 1:** Intraspecific and interspecific genetic distances of the ITS2 region for discriminating *A. montanus* from the pharmacopoeial *Acanthus* species.

Species Comparison	No. of Samples	Mean Intraspecific Distance ± S.E.	Mean Interspecific Distance ± S.E.	Barcoding Gap
*A. ebracteatus* vs. *A. montanus*	7 vs. 8	0.0131 ± 0.01/ 0.0010 ± 0.01	0.1140 ± 0.03	0.0791
*A. ilicifolius* vs. *A. montanus*	5 vs. 8	0.0322 ± 0.01/ 0.0010 ± 0.01	0.1150 ± 0.11	0.0595

**Table 2:** Transfer helix similarity of predicted ITS2 secondary structures for discriminating *A. montanus* from pharmacopoeial *Acanthus* species.

Haplotype	Transfer Helix 1	Transfer Helix 2	Transfer Helix 3	Transfer Helix 4
*A. ebracteatus*				
1	95.238	91.667	93.750	0.000
2	66.667	75.000	79.487	33.333
3	95.238	91.667	93.750	0.000
*A. ilicifolius*				
4	95.238	91.667	90.625	0.000
5	66.667	83.333	71.795	33.333
*A. montanus*				
6	66.667	100.000	100.000	30.000
7	66.667	100.000	100.000	40.000
8	66.667	100.000	100.000	40.000

**Table 3:** Collection localities, GPS coordinates, and GenBank accession numbers of *Acanthus ebracteatus*, *A. ilicifolius*, and *A. montanus* samples used in this study.

Plant Specimens	Code	Locality (District, Province, GPS Coordinates)	Accession Number
*A. ebracteatus*	NPM01	Sattahip, Chonburi (12.703529, 100.838668)	LC646039
	NPM02	Si Banphot, Phatthalung (7.664773, 99.838232)	LC646052
	NPM03	Mueang Nakhon Pathom, Nakhon Pathom (13.818120, 100.039432)	LC646055
	NPM04	Sai Noi, Nonthaburi (14.044725, 100.309735)	LC646071
	NPM05	Kantang, Trang (7.405736, 99.521326)	LC940014
	NPM06	Mueang Songkhla, Songkhla (7.154485, 100.568887)	LC940015
	NPM07	Pho Sadet, Nakhon Si Thammarat (8.413840, 99.960116)	LC940016
*A. ilicifolius*	NPM08	Mueang Trat, Trat (12.210619, 102.551772)	LC646051
	NPM09	Mueang Trang, Trang (7.568996, 99.598873)	LC646054
	NPM10	Kura Buri, Phang Nga (9.047164, 98.409568)	LC656056
	NPM11	Pho Sadet, Nakhon Si Thammarat (8.416654, 99.953591)	LC940017
	NPM12	Mueang Nakhon Si Thammarat, Nakhon Si Thammarat (8.428053, 99.956002)	LC940018
*A. montanus*	NPM13	Mueang Chiang Mai, Chiang Mai (18.790333, 98.966401)	LC646050
	NPM14	Kuan Kanun, Phattalung (7.736921, 100.006556)	LC646053
	NPM15	Sangkha, Surin (14.462985, 103.763479)	LC646070
	NPM16	Sainoi, Nonthaburi (14.044725, 100.309735)	LC940019
	NPM17	Khiri Rat Nikhom, Surat Thani (9.018175, 98.969843)	LC940020
	NPM18	Kantang, Trang (7.406373, 99.518805)	LC940021
	NPM19	Mueang Chiang Mai, Chiang Mai (18.768073, 98.941431)	LC940022
	NPM20	Mae Rim, Chiang Mai (18.888644, 98.858905)	LC940023

## Data Availability

The original contributions presented in this study are included in the article/Supplementary Materials. Further inquiries can be directed to the corresponding author.

## Supplementary Materials

The following supporting information can be downloaded at: https://www.mdpi.com/article/10.3390/plants15152349/s1, Table S1: Variable nucleotide sites and ITS2 haplotype patterns among *A. ebracteatus*, *A. ilicifolius*, and *A. montanus*.

## Abbreviations

The following abbreviations are used in this manuscript:

DAD	Diode-array detection
DNA	Deoxyribonucleic acid
ESI	Electrospray ionization
GC–MS	Gas chromatography–mass spectrometry
HMM	Hidden Markov model
ITS2	Internal transcribed spacer 2
LC–QTOF/MS	Liquid chromatography–quadrupole time-of-flight mass spectrometry
MS	Mass spectrometry
MS/MS	Tandem mass spectrometry
NJ	Neighbor-joining
PCA	Principal component analysis
PCR	Polymerase chain reaction
PC	Principal component
PLS-DA	Partial least squares discriminant analysis
ppm	Parts per million
QTOF	Quadrupole time-of-flight
S.E.	Standard error
TCS	Templeton–Crandall–Sing
UHPLC	Ultra-high-performance liquid chromatography
UPLC	Ultra-performance liquid chromatography
UV	Ultraviolet
VIP	Variable importance in projection
