# Supplementary material for: Integrated ITS2 DNA Barcoding and LC–QTOF/MS Metabolomics for Authentication of Ngueak Pla Mo (Acanthus spp.) and Differentiation of Pharmacopoeial Species from A. montanus L
Source: Plants (Basel). 2026 Jul 30;15(15):2349. doi: 10.3390/plants15152349 (PMC13468589; doi:10.3390/plants15152349)
Supplement: Supplementary file 1 [file plants-15-02349-s001.zip › plants-4440166-supplementary.pdf]

Integrated ITS2 DNA Barcoding and LC–QTOF/MS Metabolomics for Authentication of Ngueak Pla Mo (*Acanthus* spp.) and Differentiation of Pharmacopoeial Species from *A. montanus* L.

Siripat Chaichit [1], Ampai Phrutivorapongkul [1], Warunya Arunotayanun [2], and Aekkhaluck Intharuksa [1,*]

[1] Department of Pharmaceutical Sciences, Faculty of Pharmacy, Chiang Mai University, Chiang Mai 50200, Thailand; siripat.chaichit@cmu.ac.th (S.C.); ampai.phrutiv@cmu.ac.th (A.P.)

[2] Kanchanabhishek Institute of Medical and Public Health Technology, Faculty of Public Health and Allied Health Science, Praboromarajchanok Institute, Nonthaburi 11150, Thailand; warunya@kmpht.ac.th

* Correspondence: aekkhaluck.int@cmu.ac.th

Table S1: Variable nucleotide sites and ITS2 haplotype patterns among *A. ebracteatus*, *A. ilicifolius*, and *A. montanus*.

Samples	17-26	29	32	41-54	78	101	107-8	114	118	127	130	142	147-9	153-161	167	169-70	176-77	188	201-4	209	217	219	225	227-9	231	Length (bp)
Acanthus ebracteatus																										
**Haplotype 1 (NPM01, 03)**	CTCTCCCCG	C	A	CGGCCTTGTGGCGG	G	C	AT	C	T	T	C	G	ACG	AGCCCGCGC	G	GC	TT	-	CG-G	-	G	G	T	CGT	CGC	230
**Haplotype 2 (NPM02)**	*********	*	*	************-*	*	A	*G	*	*	*	T	*	***	********T	C	**	**	-	AC-*	-	*	*	*	***	***	229
**Haplotype 3 (NPM04-07)**	*********	*	*	************-*	*	*	*G	*	*	*	*	*	***	********T	C	**	**	-	AC-*	-	*	*	*	***	***	229
Acanthus iliciflolius																										
**Haplotype 4 (NPM08-09)**	*********	*	*	************-*	*	*	*G	*	*	*	T	C	***	********T	C	**	**	-	**-*	-	*	*	*	***	***	229
**Haplotype 5 (NPM10-12)**	*C*******	*	*	******C*G***--	*	*	*G	T	*	A	*	*	***	*********	C	T*	*C	-	**-*	-	*	*	*	***	***	228
Acanthus montanus																										
**Haplotype 6 (NPM13)**	---------	T	C	-------------A	T	*	GG	*	C	*	*	*	GAC	G--------	C	*T	AC	C	**C*	G	A	C	C	AAG	GCG	202
**Haplotype 7 (NPM14)**	---------	T	C	-------------A	T	*	GG	*	C	*	*	*	GAC	G--------	C	*T	AC	C	**-C	-	A	C	C	A*G	***	200
**Haplotype 8 (NPM15-20)**	---------	T	C	-------------A	T	*	GG	*	C	*	*	*	GAC	G--------	C	*T	AC	C	**-C	-	*	C	C	A*G	***	200
